# Anesthetic Management and Considerations During Surgical Dissection of a Schwannoma Causing Severe Cervical Spinal Canal Stenosis and Vertebral Artery Compression

**DOI:** 10.7759/cureus.72307

**Published:** 2024-10-24

**Authors:** Laurel Hergenroeder, Chris King, Arpan Kohli

**Affiliations:** 1 Anesthesiology, West Virginia University School of Medicine, Morgantown, USA

**Keywords:** cervical vertebrae, mean arterial pressure, neuroanesthesiology, neuromonitoring, surgical dissection, vertebral arteries, vertebral artery compression, video laryngoscopy (vl)

## Abstract

Neurosurgical cases require meticulous anesthetic planning and execution by the anesthesiologist. This report aims to illuminate the careful considerations required in several aspects of anesthetic management, including spinal positioning during intubation/throughout the case, neuromonitoring, and anesthetic agent selection to ensure adequate neural tissue perfusion and optimal outcomes in neuroanesthesia cases. We describe the anesthetic case of a large cervical spine schwannoma resection in a 64-year-old woman who experienced various neurological symptoms due to this mass. MRI revealed that the mass was causing severe spinal canal stenosis, jugular vein effacement, and compression of the vertebral artery. This patient, classified as an American Society of Anesthesiologists (ASA) III, was optimized in preoperative clinic visits. She was deemed an appropriate candidate for a posterior cervical spine laminectomy with tumor resection and instrumentation which was recommended by both Otolaryngology and Neurosurgery. Intraoperatively, a video laryngoscopy was performed to limit extreme cervical spine movement during intubation. To allow for continuous neuromonitoring throughout the case, intravenous infusions of propofol and remifentanil were the primary anesthetic along with half a minimum alveolar concentration of sevoflurane to avoid intraoperative awareness. No major neurological changes were noted during the case. Additionally, ASA standard monitoring, an arterial line, and a Bispectral Index (BIS) monitor were utilized. All anesthetic agents were titrated to achieve optimal blood pressure and BIS readings. The surgery was completed successfully and the patient did not require transfusion of any blood products. She was successfully extubated and transferred to the neurocritical care unit with no postoperative complications.

## Introduction

Neurosurgical procedures can pose high risks and thus, many aspects of the anesthetic plan must be carefully considered. Additional monitoring and safeguards, including intubation and positioning considerations as well as neuromonitoring, should be utilized, especially when pathology involves the cervical spine. Most neurological injuries during anesthesia are due to prolonged deformation of the spinal cord, impaired perfusion of the spinal cord, or both [1]. Even minor compression or stretching of the spinal cord, if prolonged, can lead to hypoperfusion of the cord and neurological damage [2]. Therefore, it is crucial to minimize cervical spine movement.

Utilizing video laryngoscopy should be considered for intubation as opposed to direct laryngoscopy, which may require more cervical spine movement. In the case of an especially difficult intubation, a fiberoptic bronchoscope may be used. Cervical spine stabilization is also crucial during prone positioning and throughout the procedure to avoid further insult to the neural tissue and ensure proper perfusion [3]. Proper titration of anesthetic agents and pressor support to maintain mean arterial pressures (MAPs) within a specified range are also crucial to ensure adequate spinal cord perfusion pressure [4]. 

Neuromonitoring during surgical manipulation of the spinal cord is essential to detect unintentional deformation or injury to neural tissue [5]. Somatosensory evoked potentials (SSEPs) and motor evoked potentials (MEPs) may be continuously monitored to surveil the integrity of both the sensory and motor tracts of the spinal cord, respectively.

We describe a case of a resection of a recurrent schwannoma in the cervical spine and highlight intubation, neuromonitoring, anesthetic maintenance and titration, and positioning considerations.

## Case presentation

A 64-year-old female patient presented to the neurosurgical clinic with a right-sided neck mass that was causing a myriad of symptoms. She experienced right hand swelling and numbness as well as shaking with activity. Additionally, she noted numbness in her posterior neck/shoulders, headache, lightheadedness, pain with neck movement, frequent falls, and occasional dysphagia. Notably, she had undergone resection of a right cervical schwannoma seven years prior to presentation.

**Figure 1:**
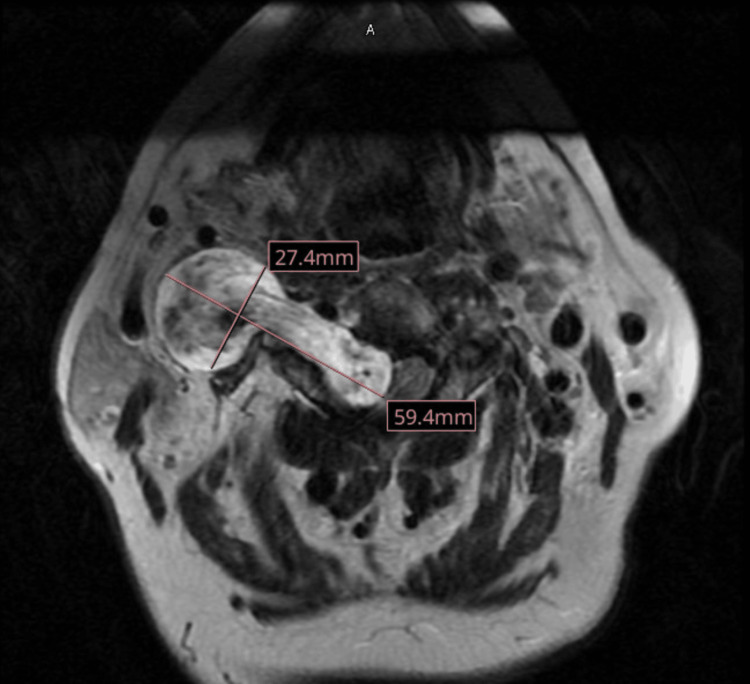
T2 weighted MRI image of schwannoma showing spinal canal stenosis and vertebral artery compression.

MRI shown in Figure 1 revealed a large dumbbell-shaped mass located at the right C2-C3 neural foramen, extending into the deep spaces of the neck. This mass caused severe spinal canal stenosis, jugular vein effacement, and compression of the vertebral artery, which can be seen in the MRI. To address this complex case, a 10-hour combined ENT and neurosurgical posterior cervical spine laminectomy with tumor resection and instrumentation was recommended and scheduled.

Preoperative visits were completed by our preoperative clinic, ENT, and Neurosurgery to confirm that this patient was an appropriate candidate for the proposed procedures. Nutritional status, hepatic function, complete blood count, basic metabolic panel, coagulation, and renal function were evaluated. Careful consideration to optimize her medical diagnoses was taken, which included gastroesophageal reflux, hiatal hernia, essential hypertension, hyperlipidemia, and sleep apnea, and she was classified as American Society of Anesthesiologists (ASA) class III. Medications prescribed by her primary physician were confirmed to be optimized and all symptoms were controlled. Flexible laryngoscopy was completed prior to surgery to visualize any lesions, masses, or other abnormalities in the airway and none were found. Her only known allergies were to sulfonamides and penicillins, resulting in skin rash only. She weighed 121 kg on the day of the surgery.

On preoperative anesthesia evaluation on the day of surgery, she was classified as Mallampati class II with full neck range of motion. She was given midazolam 2 mg in the preoperative area. For anesthetic induction, the patient received intravenous fentanyl 100 mcg, lidocaine 70 mg, and propofol 240 mg. For paralysis during intubation, she received succinylcholine 100 mg. Sevoflurane 2% was used during mask ventilation to ensure a deep plane of anesthesia prior to intubation.

To achieve successful intubation, we ensured sufficient preoxygenation and denitrogenation by mask ventilating with 100% oxygen until an end-tidal oxygen concentration was above 80%. In order to avoid further spinal cord damage and neurologic insults, it was crucial to limit sustained cervical spine movement throughout the case. C-spine stabilization was performed along with video laryngoscopy with the most experienced anesthesia provider and were able to achieve intubation with minimal cervical spinal movement. The patient was then connected to a ventilator on the volume control setting.

For maintenance of anesthesia, a propofol infusion was started at 150 mcg/kg/minute. For analgesia, a remifentanil infusion was started at a rate of 0.15 mcg/kg/minute (total 3,876 mcg). Half of a minimum alveolar concentration (MAC) of sevoflurane was used to decrease the risk of intraoperative awareness and to minimize effects on neuromonitoring. Only phenylephrine and ephedrine were required as pressor support. No paralytic was used after intubation in order to allow for neuromonitoring as a paralytic would inhibit the transmission of MEPs. Standard ASA monitoring was used as well as an arterial line in the left radial artery. Additionally, a Bispectral Index (BIS) monitor was used to ensure adequate sedation. All maintenance medications were titrated to maintain MAPs between 85-90 mmHg and the BIS value between 40 and 60. Continuous ASA monitoring was recorded on the anesthesia report.

During surgical dissection, neuromonitoring was utilized to continuously assess the integrity of the patient's spinal cord tissue. Somatosensory evoked potentials as well as motor evoked potentials were watched closely by neuromonitoring technicians present in the room during surgery. No significant changes were noted by the technicians throughout the procedure.

During the case, no surgical or anesthetic complications were encountered. The estimated blood loss was 1700 mL and no blood products were administered. The patient was fluid resuscitated with normal saline 1 L, lactated ringers 700 mL, and Plasma-Lyte 2 L (Baxter International Inc., Deerfield, Illinois, United States). At the end of the case, the remifentanil and propofol infusions as well as sevoflurane were stopped to allow for emergence. The patient was successfully extubated while still deeply anesthetized in order to avoid sympathetic response and hypertension. The patient was then transferred to the neurological critical care unit with no postoperative complications.

## Discussion

Due to the complexity of this case involving a tumor causing severe cervical spinal canal stenosis and vertebral artery compression, meticulous airway management, patient positioning, neuromonitoring, and hemodynamic control were of utmost importance.

The vertebral arteries play a pivotal role in providing blood supply to the posterior brain and spinal cord and may be compressed with cervical spine movement. In our case, the patient’s tumor was already compressing the vertebral arteries. Most cervical spine movement during direct laryngoscopy is concentrated in the upper c-spine with minimal movement below C3 [6]. Additionally, the spinal cord is most likely to suffer ischemic events at the level of C2-C3 [7]. Considering the pathology in this patient was within the levels of C2-C3, performing a video laryngoscopy was essential to reduce cervical spinal movement to retain spinal cord perfusion and avoid further compression and/or injury.

Prone positioning without cervical spine stabilization can compress the neck veins and arteries and reduce venous return, resulting in decreased preload which can lead to hypoperfusion resulting in severe neurological compromise and stroke [8]. During cases such as ours, it is extremely important to maintain patency of the vertebral arteries with its already compromised flow due to tumor location. In the present case, this was achieved by carefully stabilizing the cervical spine while moving the patient into the prone positioning as well as throughout the entire case.

During the surgical dissection, we also closely monitored the patient with neuromonitoring of the dorsal columns, using SSEPs, and anterior horn motor neuron function, using MEPs. SSPEs should not be used as the sole neuromonitoring modality as changes during surgery are not always associated with postoperative neurological deficits [9]. Additionally, ischemic changes may take 2-34 minutes to be reflected by SSPEs [10]. MEP monitoring is the gold standard for intraoperative changes but is sensitive to anesthetics and neuromuscular blockades [9]. As these two neuromonitoring modalities monitor different spinal cord tracts and have various benefits and deficits, it is important to use them simultaneously. Inhaled anesthetics like sevoflurane and desflurane inhibit SSEPs and MEPs if used at a primary anesthetic concentration (ie. a MAC of ≥1). To avoid intraoperative awareness in the patient, approximately 0.5 MAC is used which still allows for neuromonitoring signals to be observed [11].

Maintaining MAPs is beneficial for patients with spinal cord pathology [1]. We maintained MAPs between 85-90 via titration of phenylephrine and ephedrine to ensure perfusion of neural tissue while avoiding hypertension.

All aspects of the anesthetic plan during neurosurgical cases require special consideration to avoid further neurological insults. Careful consideration of anatomy and the location of pathology and surgical manipulation should guide laryngoscope modality, patient positioning, monitoring modalities, anesthetic agents, and physiologic ranges to which anesthetics should be titrated.

## Conclusions

Neurosurgical procedures pose a high risk of complication, especially when there is pre-existing spinal cord compression or risk of ischemia. However, a vigilant anesthesiologist can help decrease the risk with careful consideration when formulating and executing the anesthetic plan. Spinal movement should be kept to a minimum and maintenance of neural tissue perfusion should be a priority. These goals can be achieved via video laryngoscopy, cervical spine stabilization during prone positioning, multimodal neuromonitoring, and careful anesthetic titration.

When managing anesthesia during a case involving cervical spine pathology, it is crucial to restrict movement to this region. This can be done via video laryngoscope or fiberoptic bronchoscopy for more difficult intubations. Neuromonitoring allows for real-time feedback regarding the integrity of motor and sensory tracts to avoid neurological damage during surgical procedures involving the spine. Utilization of inhaled anesthetic gasses at the concentration required for general anesthesia can diminish neuromonitoring signals. Therefore, it is beneficial to use intravenous anesthetic infusions such as propofol and remifentanil as they do not inhibit these signals at higher concentrations. Consideration of the physiological impacts of each patient’s unique pathology allows the anesthesiologist to make adjustments that improve the anesthetic efficiency, overall surgical case, and patient outcomes.
